# Skill mix in Swiss primary care group practices - a nationwide online survey

**DOI:** 10.1186/s12875-019-0926-7

**Published:** 2019-03-04

**Authors:** Renata Josi, Carlo De Pietro

**Affiliations:** University of Applied Sciences of Southern Switzerland, Department of Business Economics, Health and Social Care, Via Violino 11, Manno, 6928 Switzerland

**Keywords:** Primary care, Personnel composition, Advanced roles, Advanced roles

## Abstract

**Background:**

Increasing chronic conditions and multimorbidity is placing growing service pressures on health care, especially primary care services. This comes at a time when GP workforce shortages are starting to be felt across Switzerland, placing a threat on the sustainability of good access to primary care. By establishing multiprofessional teams in primary care, service capacity is increased and the pressures on the GP workforce can be alleviated. The roles of non-medical health professions in primary care are not established so far in Switzerland and the personnel composition of primary care group practices is not known. Therefore this study aims to provide insights into the current composition, educational background and autonomy of the these new professional roles in primary care.

**Methods:**

For this descriptive exploratory study a web-based online survey methodology was used. Group practices were defined as being a medical practice with any specialisation where at least three physicians work together in a team. Based on this restriction 240 eligible group practices were identified in Switzerland. The following four tertiary-level health professions were included in the study: nurses, physiotherapists, occupational therapists and dietitians. Additionally medical practice assistants with couselling competencies were included.

**Results:**

A total of 102 practices answered the questionnaire which is equivalent to an answer rate of 43%. The sample included data from 17 cantons. 46.1% of the practices employed non-physician health professionals. Among the tertiary-level health professions, physiotherapists were the most frequent profession with a total of 78 physiotherapists over all group practices, followed by nurses (43), dietitians (34) and occupational therapists (3). In practices which employ those professionals their average number per practice was 3.4. 25.5% of the practices had health professionals employed with advanced roles and competencies.

**Conclusion:**

The results from this study demonstrate that while nearly 50% of groups practices have established non-physician professionals, only 25% of practices integrate these professionals with advanced roles. Compared with other countries, there would appear to be significant scope to extent and broaden the uptake of non-physician professionals in primary care in Switzerland. Clear policy direction along with supporting regulation and financing arrangements are required.

**Electronic supplementary material:**

The online version of this article (10.1186/s12875-019-0926-7) contains supplementary material, which is available to authorized users.

## Background

In Switzerland, as elsewhere, the ambulatory care sector (including primary care) is challenged by the ageing of the population, as well as the rapid increase in the number of patients suffering from chronic diseases [1, 2]. These epidemiological changes will, in the future, significantly increase the demand for health care services. Unlike other sectors, in the health sector, the increasing demand for services is not yet accompanied by an equally rapid increase in workforce, especially physicians and other health professionals. The capacity of medical schools, as well as of educational programs for other health professions, has been increased but they are still not yet educating a sufficient number of medical doctors and other health professionals in Switzerland [3, 4].

The Swiss primary care sector mainly consists of general practitioners (GPs) working in individual private practices and usually helped by medical practice assistants which are educated at higher vocational schools for three years and are mainly involved in administrative tasks (front desk, billing ect). The sustainability of this traditional arrangement is under threat, given the shortages of physicians available to work in these practices [5]. Along these dominant individual practices, in recent years there has been a strong development of group practices. These practices often enable several medical specialties to be offered in one place thus facilitating more integrated and coordinated care. The increase of group practices also reflects the emerging preference of physicians for new working models which allow for a better work-life balance. In Switzerland, many single practices, as well as group practices, are part of a professional network which supports medical activities by providing shared administration or organizing further education of the participating physicians.

It is noteworthy that the composition and skill mix of group practices vary significantly, within Switzerland and internationally [6–8]. There are many countries where the median number of non-physician health professionals per primary care practice is substantially higher than in Switzerland. For example, in European countries such as England, Finland, Lithuania, the Netherlands, Portugal, Spain and Sweden at least three other non-physician professionals are employed in primary care practices. A similar situation is also observed in some non-European countries such as Australia and Canada. In the USA, Canada, the UK and the Netherlands nurses are the largest non-physician workforce in primary care. Whereas in Austria, Germany, Luxemburg and Switzerland, medical practice assistants are the main non-physician workforce in general practice [8]. In addition to differences in skill mix, the educational programs for the different professions practicing in primary care differ significantly between countries [7]. These differences illustrate that countries have found different ways of preparing and organizing their primary care workforce in seeking to satisfy the ever more complex needs of their patients.

Patients’ needs are becoming ever more complex due to the increase in the prevalence of chronic diseases and multimorbidity. This situation is now creating a higher health care burden on higher-income countries such as Switzerland. In 2011, non-communicable diseases (NCD’s) were responsible for 80% of the total costs of the health system of Switzerland [9]. In 2012, the Swiss Health Survey found that 31.9% of the Swiss population older than 15 years are affected by at least one chronic disease [10]. In the 2010/2011 Survey of Health, Ageing and Retirement in Europe (SHARE) 49.9% of the Swiss population older than 50 years said that they suffer from at least one chronic disease, with 22% of these people reporting they are affected by more than one disease [10]. The Swiss Health Observatory has projected that in order to cope with the increasing demand, GP workforce will need to increase by up to 40% by 2030, compared to the 2004 level [11]. However, the GP workforce is aging, with the mean age of doctors working in the primary care sector in Switzerland at 54.8 years in 2017 [12], 2 years older on average than in 2008 [13]. In 2015, it was projected that nearly half of the primary care doctors of Switzerland will have reached retirement age by 2022, leading to a shortage of primary care doctors [10].

There is an ongoing policy dialogue in Switzerland on how to address GP workforce shortages [14–16]. International experience suggests that the integration of trained nurses into primary care teams, along with GPs and other health professionals can play a role in alleviating physician workforce pressures and improve care and management of chronic care needs in the community [17–19]. According to Green (2013) [20] the shortage of primary physicians could be nearly eliminated through the efficient integration of allied health professionals into primary care practices. The international literature indicates that the integration of specially trained nurses into primary care teams can enable nurses to safely and effectively carry out roles and fulfill certain tasks usually undertaken by physicians. These roles are more generally referred to as "Advanced Nursing Practice (ANP)" and are already being widely implemented in other countries such as Australia, Ireland, UK and the US [19, 21]. In Switzerland, the uptake of ANP is largely confined to the inpatient care setting, with commensurate roles in the primary care sector not well defined or established at this time. Although much discussion revolves around ANP and a greater role for the nursing profession in primary care, there is also expanding consideration being given to the advanced roles that other health professionals such as physiotherapists, occupational therapists, pharmacists and dietitians can play in the ambulatory care setting [22]. In addition, over the last few years, it has become possible for medical practice assistants in Switzerland to obtain further education qualifications (either in chronic care management or practice management) to acquire the skills to counsel patients with chronic diseases following a structured checklist.

The degree to which non-physician health professionals are involved in primary care practices has not been investigated on a national level so far in Switzerland. Therefore, the principal objective of this study was to identify and characterize the current workforce and skill mix of Swiss group practices from a representative sample of primary care practices. The focus was on the presence of 4 non-physician tertiary-level health professions: nurses, physiotherapists, occupational therapists, and dietitians. Tertiary-level in this context means that professionals are educated at universities or at universities of applied sciences and hold a bachelor’s or master’s degree. The secondary objectives of our study were to a) investigate the specific educational backgrounds of these 4 tertiary-level health professionals working in the group practices and b) identify if these health professionals work in advanced roles and have advanced competencies in practice. For the purposes of this study, the following definition of advanced roles was used: “Advanced roles refer to roles that contain one or more of the following areas of responsibility: Management of other employees, mentoring and coaching of employees, research activities, implementation of research results in practice, preparation of guidelines, care and management of complex patients, performance of assessments and clinical expertise”. This definition is based on the international definition of advanced practice by the international council of nurses [23]. Our objectives translated into the following two research questions:

What is the current skill-mix in Swiss primary group practices of physicians, medical practice assistants with couselling competencies and the four health professions nursing, physiotherapy, dietetics and occupational therapy?

Which educational degrees have tertiary-level health professionals who work in primary care group practices in Switzerland obtained and do they work in advanced roles with advanced competencies?

## Methods

### Study design and sample

This study is exploratory, given the preliminary nature of existing research into the general practice workforce in Switzerland. We use the Swiss Federal Office of Public Health’s terminology and refer to certain health professions by using the term “tertiary-level health professionals”. Tertiary-level in this context means that professionals are educated at universites or universities of applied sciences and hold a bachelor’s or master’s degree. We focused on four tertiary-level health professions (Nursing, Physiotherapy, Occupational Therapy and Dietetics) in this study, given they are (together with midwifes, optometrists and osteopaths) regulated in Switzerland under the Act on Health Professionals (GesBg) which came into effect during 2016 [24]. In addition to GPs and medical specialists, medical practice assistants - but only those with further education and counselling competencies - were also included in the scope of our study. Medical practice assistants are health professionals educated at higher vocational schools for three years who then work in primary care practices. Their scope of practice mainly involves administrative tasks but they can with limited competencies after having obtained further education counsel patients with chronic diseases under the supervision of a physician. We included also this occupational group because it actively contributes to the management of chronic diseases and other conditions in primary care.

To create the sample of group practices, 240 group practices in Switzerland were identified through the Forum Managed Care, an organization which fosters the exchange of knowledge and experiences with integrated care in Switzerland. Because there are no official databases on group practices in Switzerland, the Forum Managed Care which is a network of healthcare providers, patients, insurers, politicians and other stakeholders was the only organisation which could provide reliable and representative information on group practices in Switzerland. The organization was able to provide the details of 7 operating companies that manage 61 physician networks and provide administrative services. These 7 operating companies plus a further eight practice networks without an operating company were contacted by telephone by the research team during the 3 month period February to April 2018. To be an eligible group practice, the practice had to have at least three physicians working together in a team, regardless of their specialisation. As there exists no single standard empirical definition of a group practice, the authors considered group practices with three or more physicians working together. In fact, a double practice consisting of two doctors can hardly be considered a group and it is often a minimal organizational arrangement useful to share one medical practice assistant or to guarantee the openness of the practice throughout the year. On the other side, a threshold higher than three would have restricted too much the sample. The sampling was not restricted to practices with a specialisation in internal medicine or family medicine because Switzerland has no gate-keeping system and primary care services can therefore be provided also by specialists or in group practices where both family doctors and specialists work together. No other criteria was specified for the sample selection. The sample is considered nationally representative as all existing operating companies and several practice networks in Switzerland were considered and contacted.

### Data collection

The principal method of data collection for this research involved a web-based survey. The survey was disseminated to the 240 group practices by the operating companies and practice networks or, where this was not possible, via initial telephone contact and subsequent e-mail by the research team. A reminder to complete the survey was sent to non-responding group practices four weeks after initial dissemination. While the responses were anonymous, participant group practices had to register online to get a unique token to complete the survey. This provided the research team the capacity to identify group practices to contact to remind them to complete the survey. There was no financial incentive for practices to fill in the survey.

### Questionnaire

The questionnaire was created online using LimeSurvey to collect data on the composition and educational background of the workforce in Swiss group practices. The questions were first created in German as this is the most spoken language in Switzerland and the mother tongue of the first author. The items were developed through discussion among authors. The study aims research questions lead the authors to clearly defined questions to be asked through the questionnaire to be able to answer the research questions.

As the sample included practices from all parts of Switzerland, the questionnaire was also translated into French and Italian. The questionnaire required completion of between six and sixteen questions, depending on the answers given to preceding questions. Further details on the questionnaire are provided at Additional file 1: Table S1. The French and Italian translation involved forward-backward translation by two health scientists and two professional translators respectively. The original German version was compared to the two back-translations. While no major inconsistencies were detected during this process, minor changes were subsequently made to the questionnaire by the research team. The questionnaire was validated in all three languages by five people working in the area of health at the University of Applied Sciences of Southern Switzerland. They were asked to assess the comprehensibility and the reliability of the questionnaire. Minor changes were made by the research team upon review of the validation feedback.

### Data analysis

The data from completed responses was imported from the LimeSurvey database to STATA and Microsoft Excel for the analysis. Descriptive statistics were generated for key variables including: the number of medical and non-medical personnel, educational degrees of tertiary-level health professionals, occupancy rate and whether or not the professionals worked in advanced roles with advanced competencies.

## Results

The response rate to the survey was 43%, with a total of 103 complete responses provided by group practices through the online survey facility. One response was excluded due to data quality issues (i.e. implausible responses to questions – for example: reporting 70 nurses participating in the group practice). This response could not be validated as the answers were anonymized. In most cases the questionnaire was filed in by the medical practice assistants who have full administrative information about the medical practices. In addition to the responses coming from group practices we have received four responses from practices with only two physicians and one answer from a practice with one physician. Although these five answers did not meet the eligibility criteria of a group practice the research team decided to keep the in the sample for the analysis. These five practices had involved one or more health profession of interest for this study in their practice and were therefore considered to provide valid data about the workforce composition of general practices in Switzerland. Apart from the number of doctors in the practice their answers did not differ from the answers of those practices where more doctors collaborated. The final sample consisted of 102 practices.

The sample included practices operating in 17 different cantons, which represent 65 percent of the total cantons and 90% total population coverage in Switzerland. Table 1 summarizes the main practice characteristics of the sample. Over two thirds of the group practices were part of a practice network with various practices located at different places within and across the cantons (68.6%) and nearly half of the practices employed tertiary-level health professionals in their practice (46.1%).

**Table 1:** Characteristics of group practices (more than one answer admitted)

Variable	N	%
Practice is part of a practice network	70	68.6
Practice has MPA’s *	63	61.8
Practice has health professionals**	47	46.1
Practice has health professionals** with advanced roles	26	25.5
Total	102	100

*N*otes: * Medical practice assistants with further education to counsel patients with specific diseases. ** includes Nurses, Physiotherapists, Occupational therapists and Dietitians

### Doctors

The number of doctors working in the group practices ranged from 1 to a maximum of 49 doctors per practice. This latter data came possibly from practice networks where the there was only one questionnaire answered for several primary care practices which operate under one roof, adding the number of doctors up. The mean number of doctors was 9 as shown in Table 2. Table 3 shows that more than half of the practices (56.9%) employed less than six doctors. The average number of doctors was the highest in the canton of Geneva with 13.5 doctors and the lowest in the canton of Aargau with 3.8 doctors. Nearly all practices (91%) were general practices offering internal medicine. Other medical specialties offered in group practices were Gynecology and Obstetrics (28.4%), Pediatric and adolescent medicine (28.4%) and Psychiatry and Psychotherapy (23.5%). For a detailed list of all medical specialties offered see Additional file 2: Table S2.

**Table 2:** Personnel composition per group practice sorted by Canton

Canton	Number of practices	Average number of doctors	Average number of MPA* (N Practices)	Average number of Health professionals** (N Practices)
ZH	26	9.2	7.47 (17)	2.4 (10)
BE	20	10.3	7.0 (16)	5.75 (8)
GE	8	13.5	4.5 (2)	4.125 (8)
LU	7	9.2	5 (6)	3.0 (2)
SZ	6	7.3	6.0 (3)	1.3 (3)
SO	6	6.1	3 (2)	1.0 (2)
TI	5	7.6	9.667 (3)	5.0 (3)
AG	4	3.8	3.5 (2)	2.0 (1)
SG	4	5.8	6.667 (3)	8.0 (2)
BL	3	4.0	1 (1)	2.0 (1)
TG	3	12	2.3 (3)	3.0 (2)
ZG	3	6.6	2.0 (2)	2.0 (3)
BS	2	10.0	2.5 (2)	0 (0)
VD	2	17	0 (0)	4.5 (2)
FR	1	7.0	0 (0)	0 (0)
GR	1	5.0	1 (1)	0 (0)
UR	1	12	0 (0)	0 (0)
Total	102	9.03	5.95 (63)	3.36 (47)

*N*otes: * Medical practice assistants with further education to counsel patients with specific diseases. ** includes Nurses, Physiotherapists, Occupational therapists and Dietitians. The calculation of the overall average number of health professionals and medical practice assistants exluded practices which did not employ those professionals respectively. The total number of practices employing medical practice assistants was 63 and respectively 47 practices employing health professionals

**Table 3:** Size of group practices

Number of doctors in the practice	Frequency	%
1-3	21	20.6
4-6	37	36.3
7-10	16	15.7
11-20	20	19.6
>20	8	7.8
Total	102	100

### Medical practice assistants with counselling competencies

**Fig. 1:**
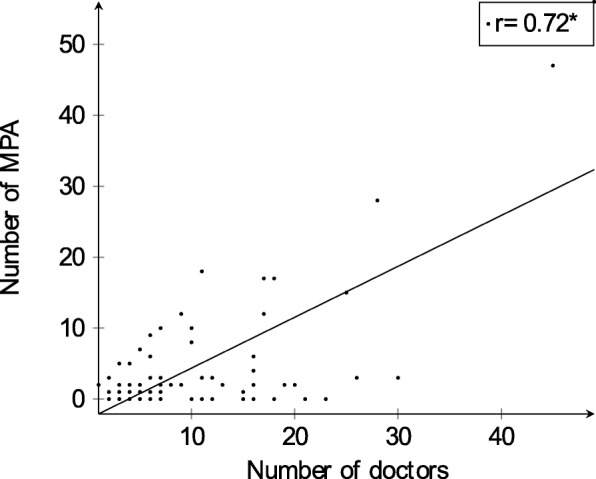
Correlation of the number of doctors and medical practice assistants

Most practices (63) employed medical practice assistants with counselling competencies. The majority of those practices (68.3%) had 1-3 medical practice assistants with counselling competencies employed. The mean number of medical practice assistants with counselling competencies per practice was nearly 6 which corresponds to 0.58 practice assistants per doctor. Figure 1 shows that there was a positive correlation between the number of doctors per practice and the number of those medical assistants per practice (r=0.72).

### Tertiary-level health professions

**Fig. 2:**
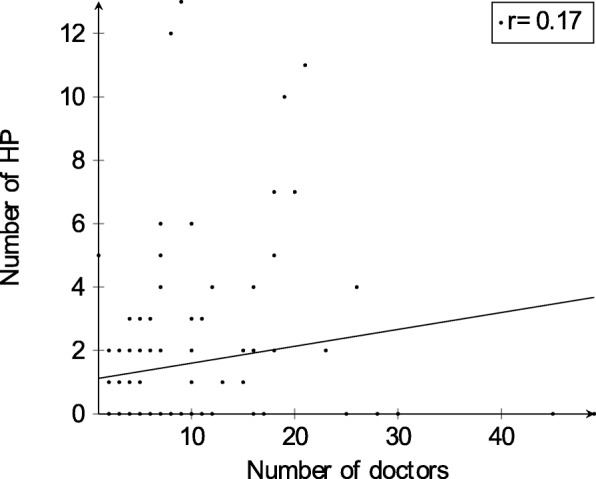
Distribution of the total number of health professionals (nurses, physiotherapists, dietitians or occupational therapists) per group practice

**Fig. 3:**
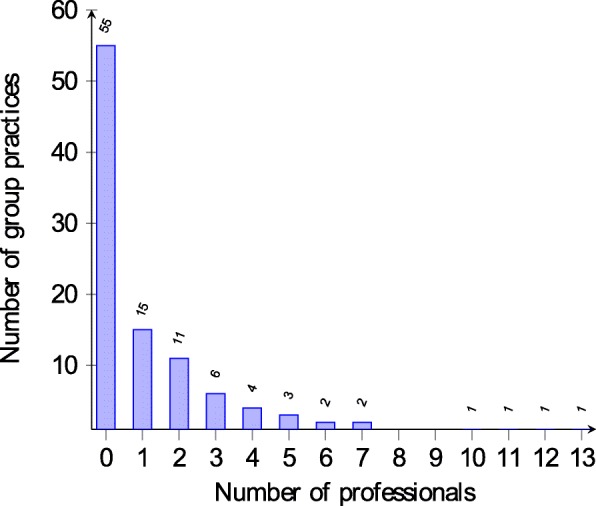
Correlation of the number of doctors and health professionals

In total there were 158 health professionals working in the participating practices and belonging to the four tertiary-level health professions considered for this analysis. Figure 2 shows that the number of health professionals per practice is right-skewed with 55 practices not employing any of those tertiary-level health professionals. Unlike practice assistants, there was no correlation (r=0.17) found between the number of doctors per practice and the number of tertiary-level health professionals (see Fig. 3). Practices in the canton of St. Gallen had the highest average of tertiary-level health professionals per practice with 8 professionals whereas four cantons (BS, FR, GR and UR) had no tertiary-level health professionals in their practices at all. The overall mean number of those four tertiary-level health professionals per practice was 3.3. 26 practices said that there were tertiary-level health professionals working in advanced roles with advanced competencies. This corresponds to 25.5% of all practices.

### Professional composition

Among the four professions in scope for this study, physiotherapists were the most numerous profession present in this sample of group practices while dietitians were the most dispersed across practices. Physiotherapists were employed in 21 practices with a total of 78 professionals. Nurses were employed in 21 practices with 43 nurses in total. Dietitians were employed in 29 practices with a total of 34 therapists. Occupational therapists were the least frequent professional group with three therapists working in three practices. Table 4 shows that among the tertiary-level health professions physiotherapists working in the same practice with dietitians was the most prevalent combination of professionals (14 practices). The combination of tertiary-level health professionals with MPA with counselling competencies was frequently occurring. Ten Practices employed Nurses and Dietitians in the same practice. Four practices employed three different health professions of which three practices worked with the combination of nurses, physiotherapists and dietitians.

**Table 4:** Number of group practices employing more than one occupational group in the practice

	MPA* (%)	Nurses (%)	Physiotherapists (%)	Occupational therapists (%)	Dietitians (%)
Combined with					
MPA	-	11 (10.8)	12 (11.8)	2 (2)	22 (21.6)
Nurses	-	-	4 (4)	1 (1)	10 (9.8)
Physiotherapists	-	-	-	1 (1)	14 (13.7)
Occupational therapists	-	-	-	-	1(1)

*N*otes: * Medical practice assistants with further education to counsel patients with specific diseases

### Degree of employment

The information about the degree of employment (in %) was not complete for all observations. This was due to the fact that not all participating practices filled in the degree of employment for all their tertiary-level health professionals. Table 5 shows that 36 physiotherapists (61%) in the participating practices work at a high degree of employment of above 60%, whereas only 42.1% of nurses and 33.4% of occupational therapists and 5.4% of dietitians work at this level. For dietitians the analysis revealed that nearly all of this workforce work below 40% of employment. Based on the 40 complete responses, the mean degree of employment of all tertiary-level health professionals corresponded to nearly 2 full-time equivalents staff per practice.

**Table 5:** Degree of employment and educational degrees of health professionals

	Nurses (%)	Physiotherapists (%)	Occupational therapists (%)	Dietitians (%)
Level of employment				
1-20%	5 (13.2)	5 (8.5)	1 (33.3)	18 (64.3)
21-40%	7 (18.4)	7 (11.9)	0	7 (18.9)
41-60%	10 (26.3)	11 (18.6)	1 (33.3)	1 (2.7)
61-80%	9 (23.7)	17 (28.8)	1 (33.3)	2 (5.4)
81-100%	7 (18.4)	19 (32.2)	0	0
*Total*	38 (100)	59 (100)	3 (100)	28 (100)
Educational degrees				
Higher vocational school	19 (35.2)	25 (28.1)	1 (25)	15 (45.5)
Higher vocational school + post-diploma course	0	9 (10.1)	0	1 (3.0)
Higher vocational school + post-diploma studies	1 (1.9)	6 (6.7)	0	0
Higher vocational school + vocational examination	4 (7.4)	1 (1.1)	0	1 (3.0)
Higher vocational school + higher qualifying examination	1 (1.8)	7 (7.9)	0	2 (6.1)
Bachelor of science	7 (13.0)	19 (21.3)	1 (25)	8 (24.2)
Master of science	4 (7.4)	6 (6.7)	1 (25)	0
PhD	1 (1.8)	2 (2.3)	0	0
Certificate of advanced studies	8 (14.8)	8 (9.0)	1 (25)	3 (9.1)
Diploma of advanced studies	3 (5.6)	2 (2.3)	0	2 (6.1)
Master of advanced studies	6 (11.1)	4 (4.5)	0	1 (3.0)
*Total*	54 (100)	89 (100)	4 (100)	33 (100)

### Qualification

The data on the qualifications of the tertiary-level health professionals were not complete for every practice. The total number of health professionals per professional group reported for this variable by some practices differed from the total number reported in other sections of the questionnaire. Factors contributing to this situation include

- information was not given for all health professionals resulting in an undercount.
- in some questionnaires more than one degree was reported per health professional rather than the only the highest degree achieved, resulting in over counting.

The data from the two questions on educational degrees were consolidated for the purposes of the analysis. Table 5 summarizes the educational degrees obtained by the tertiary-level health professionals. Nurses had predominantly nursing degrees from higher vocational schools (35%) or a bachelor’s degree in nursing (13%). Four nurses had obtained a Master’s degree and one nurse had obtained a PhD. More frequent were Certificates of Advanced Studies (CAS; corresponding to a minimum of 10 ECTS) and Master of advanced studies (MAS; corresponding to a minimum of 30 ECTS). 1 ECTS point corresponds with 25-30 h work including class lectures, home study ect. Physiotherapists as well had degrees from higher vocational schools (28.1%) or bachelor’s degrees (21.3%). As further education more often a CAS or a MAS was obtained. For the dietitians data showed that they have completed their degrees at higher vocational schools (45.5%) or have obtained bachelor’s degrees (24.2%).

### Further education qualifications

Participants were also asked which further education qualifications the health professionals with advanced roles have completed. As post-diploma course the specialisation "family centered care" was mentioned once. As CAS integration of scientific knowledge, cooperation management, clinical trial management and general clinical management were mentioned. On the level of Master’s studies and MAS the Master of Science in Nursing was named together with health promotion and prevention, educational science and public health. Among other further educations wound expertise, diabetes counselling, coordination, communication, acupuncture, psychosomatics, foot care, foot reflexology massage, process and embodiment focused psychology, breathing therapy, manual therapy, diving medicine, hypnosis, chinese medicine, advanced care planning, instruction, coaching training and clinical assessment were named.

## Discussion

This study was the first of its kind in Switzerland and its results provide an overview over the personnel composition of primary care group practices in Switzerland. It showed that physiotherapists were the most prevalent group of tertiary-level health professionals followed by nurses, dietitians and few occupational therapists. The practice composition however varied considerably between cantons. Our results show that medical practice assistants with counselling competencies are the most frequent occupational group apart from physicians in Swiss group practices. Furthermore, we found that the higher the number of doctors in one practice, the higher the number of medical practice assistants. Medical practice assistants with counselling competencies are often involved in health coaching which usually includes counselling the patient along structured checklists and providing support with symptom management. However, so far there exists only limited evidence on the effectiveness of health coaching by medical assistants for patients with chronic diseases [25–28]. While medical practice assistants are the most numerous occupational group apart from physicians in Swiss primary care and their further education to take over new roles (couselling of patients with chronic diseases) is established since 2015, other health professions are less established yet and further education of tertiary-level health professionals is not specifically tailored to advanced roles in primary care.

The four tertiary-level health professionals considered in this analysis (nurses, physiotherapists, dietitans and occupational therapists) were only employed in less than half of the practices. This result is confirmed by a survey done in 34 countries on practice composition in primary care which has found that Switzerland is among the countries with the least number of extra professions apart from physicians involved in primary care practices [8]. In addition to this study, our study not only included nurses and physiotherapists but two more health professions (dietitians and occupational therapists) as well as medical practice assistants with couselling competencies. It has been shown in other studies that the inclusion of new professional roles can lead to better access to care, better patient information, higher satisfaction, better clinical outcomes, better care quality and better health care utilization without affecting costs [29, 30]. Our study found that 25.5% of all group practices had tertiary-level health professionals employed who work in advanced roles and with advanced competencies. As health professionals are not yet widely established in primary care it is much more probable to find professionals with advanced roles in group practices with higher service volume rather than in single practices. The effectiveness of the inclusion of nurse practitioners into primary care and the substitution of doctors by nurse practitioners has been proven in a number of studies [18, 30–34]. Also for physiotherapists it could be shown that they are effective in primary care of musculosceletal disorders when patients are directly triaged to physiotherapists instead of general practitioners [35]. Furthermore a systematic review showed positive effects of dietetic interventions in primary care through dietary or clinical indicators [36]. The role and clinical effectiveness of occupational therapists has rarely been investigated in the literature up to now.

The result that 1 in 4 practices employed tertiary-level health professionals with advanced roles was surprisingly high as advanced roles such as nurse practitioners or advanced practice nurses are relatively new in the Swiss outpatient sector. There are still very large differences between countries in how advanced practice is defined and what is understood by advanced practice. Although we used an international definition of advanced practice it is possible this could have been misinterpreted and therefore our results should be adopted with caution. Overall our study finds a relatively low uptake of other professional staff in group practices in Switzerland. One study involving six European countries broadly confirms this finding concluding that there are generally low numbers of nurse practitioners and physician assistants working in primary care in Europe [7].

Evidence of the effectiveness of including these professionals in primary care is however not enough to implement these skill-mix changes in practice. There are many obstacles in clinical practice which prevent the effective inclusion of new professions into primary care. For example, there exist prerequisite infrastructure requirements to support such changes. In order to satisfy patient’s needs effectively with multidisciplinary teams, systems of support are needed such as information systems, monitoring processes, guidelines and multidisciplinary team arrangements [37]. These require investment and careful planning and implementation to support skill mix changes [38]. Notwithstanding this kind of obstacle, there still persist major concerns related to the safeguarding of safety and quality of care of task shifting from physicians to non-physician health professionals. There are concerns that inadequate regulatory arrangements and financial structures are in place to enable the right checks and balances and incentives in the system to underpin the patients interests [25]. For example, the lack of supporting regulations prescribing rights for non- physician professionals has been shown to hinder the effective implementation of skill-mix changes into practice [29]. By not broadening prescribing rights to other health professionals the continuity of care can be undermined through the need for other health professionals to refer patients back to the physician for prescription of medications. The financing system in Switzerland for the ambulatory sector is also considered to create a barrier to the implementation of new professional roles. Ambulatory care services are financed by a fee for service scheme and it has been shown that this scheme does not adequately provide for the remuneration of services provided by new professional roles in care settings [25]. Swiss health policy makers are challenged to adapt the health system and to overcome these obstacles in future in order to support an effective implementation of other health professionals into the primary care sector.

### Strengths and limitations

A strength of this study was the relatively robust response rate. Our survey achieved a response rate of 43% which can be considered high given the setting in which it was conducted. A meta-analysis of 154 sub-groups of studies found an average response rate for online surveys of 38% [39]. Generally it is difficult to motivate personnel of general practices to fill in a survey questionnaire because they have a very high workload and a heavy time constraint. The survey questions were validated and translated in three languages using forward and backward translation. The multilingual questionnaire made it possible to collect answers from nearly all cantons of Switzerland. This gives the study more strength in generalizability to the whole country. In general, the responses to the survey were credible with a few exceptions where the responses to specific questions were not considered credible or logical. A weakness of this study was the completeness and representative nature of the sample. As there was no pre-existing data existing on Swiss group practices, the authors had to identify eligible group practices via their operating companies and physician networks. Even though much effort was put into identifying the highest possible number of practices, it is likely that some group practices were missed. This could bias the results if a certain type of practice or practices from specific cantons were not included in the sample. Furthermore it is not known, what practice characteristics the non-responders had. Non-response bias could affect the results if there was a certain type of practice not answering the survey. In general the authors hypothesize that those practices which did not employ tertiary-level health professionals or considered themselves too small to be counted as group practice or to be of relevance were more likely not answering the questionnaire. If this hypothesis were true, the response rate of those practices relevant for our study would even be higher and the results more credible. Concerning the credibility of the answers there are some issues to be mentioned. The degree of employment of the tertiary-level health professionals was not filled in for every employed health professional. This affected the mean degree of employment per practice as not all health professionals are included in the calculation of this mean. Furthermore, the educational degrees achieved by tertiary-level health professionals were not filled in consequently by the responders of the questionnaire. There were cases where several degrees were achieved, one after the other and they were all counted instead of only filling in the highest degree achieved as it was asked in the questionnaire. Therefore the numbers of professionals having achieved a certain degree could be upwards biased. However those cases were rare in the sample. As a last issue, the share of practices employing health professionals with advanced roles could be upwards biased because in some cases it was answered that a professional worked in an advanced role but the further education qualifications did not match with the usually expected qualifications. The authors hypothesize that in these cases medical practice assistants with counselling competencies were counted as working in advanced roles. This could be an indication for a misunderstanding of the definition of advanced practice.

## Conclusion

Switzerland will be challenged by an increasing number of chronically ill patients as well a shortage of primary care physicians in the future. To increase the efficiency and sustainability of primary care services under these circumstances, the inclusion of non-physician health professionals into primary care practices is considered by the authors of this study to be a promising approach. In Switzerland and internationally a policy discussion is ongoing about how multidisciplinary teams should be composed and which health professionals could be efficient in tackling the future challenges that health systems are facing. The results of this study illustrate the composition of primary care practices in Switzerland and could inform policy makers when deciding who should work in multidisciplinary primary care teams and how these teams should be composed for an efficient healthcare provision. To support such a skill-mix change, stakeholders from health policy and research in clinical practice settings have to clarify the roles of these professionals as well as provide the necessary supporting regulations and financing arrangements. Furthermore, research and practice should investigate cost-effective models for task division between medical practice assistants and new roles for tertiary-level health professionals.

## Additional files

Additional file 1 Questions of online questionnaire. Description of data: Additional Table showing the questions of the online questionnaire. (PDF 27 kb)

Additional file 2 Medical specialties offered by participating group practices. Description of data: Additional table showing the medical specialties which were offered by the medical practices which participated in the online survey. (PDF 26 kb)

## Abbreviations

ANP: Advanced nursing practice
CAS: Certificate of advanced studies
ECTS: European credit transfer system
GesBG: Gesundheitsberufegesetz (act on health professionals)
GP: General practitioner
MAS: Master of advanced studies
MPA: Medical practice assistant
NCD: Non-communicable disease
SHARE: Survey of health, ageing and retirement in Europe
