# Supplementary material for: Skill mix in Swiss primary care group practices - a nationwide online survey
Source: BMC Fam Pract. 2019 Mar 4;20:39. doi: 10.1186/s12875-019-0926-7 (PMC6398248; doi:10.1186/s12875-019-0926-7)
Supplement: Supplementary file 1 — Questions of online questionnaire. Description of data: Additional Table showing the questions of the online questionnaire. (PDF 27 kb) [file 12875_2019_926_MOESM1_ESM.pdf]

Table S1 Questions of online questionnaire

Question Nr	Questions	Answer choices (simplified)	Conditions
1	Is your practice part of practice network consisting of practices at different places?	Yes/No (Y/N)	Mandatory for all
2	How many doctors do work in your practice?	Number	Mandatory for all
3	Are there medical practice assistants working in your practice who do patient counselling?	Y/N	Mandatory for all
4	How many of these practice assistants in such roles work in your practice?	Number	If Q3=Y
5	Are there health professionals working in the practice?	Y/N	Mandatory for all
6	Which professional group do they belong to and how many are there per occupational group?	Numbers per profession	If Q5=Y
7	How many health professionals work in the respective level of employment?	Number sorted by profession and level of employment	If Q5=Y
8	What is the total fulltime equivalent of all health professionals in your practice?	Number	If Q5=Y
9	How many health professionals have completed one of the following educational degrees?	Numbers sorted by profession and educational degree	If Q5=Y
10	How many health professionals have completed one of the following further education qualifications?	Numbers sorted by profession and qualifications	If Q5=Y
11	Are the health professionals practicing in advanced roles or with extended competencies?	Y/N	If Q5=Y
12	Which specialisations did the continuing education courses, which the health professionals with advanced roles completed, have?	Text box	If Q5=Y
13	You have indicated that health professionals work in your practice. Would you be willing to be at our disposal for any further data collection?	Y/N	If Q5=Y
14	Please enter below an email address and/or telephone number, where we can contact you.	Text box	If Q5=Y
15	Which medical specialties are offered in your practice?	List of all medical specialties	Mandatory for all
16	In which canton does your practice operate?	List of all Swiss cantons	Mandatory for all
