# Supplementary material for: Skill mix in Swiss primary care group practices - a nationwide online survey
Source: BMC Fam Pract. 2019 Mar 4;20:39. doi: 10.1186/s12875-019-0926-7 (PMC6398248; doi:10.1186/s12875-019-0926-7)
Supplement: Supplementary file 2 — Medical specialties offered by participating group practices. Description of data: Additional table showing the medical specialties which were offered by the medical practices which participated in the online survey. (PDF 26 kb) [file 12875_2019_926_MOESM2_ESM.pdf]

Table S2 Medical specialties offered by the 102 participating group practices

Medical specialties	N	%
Internal Medicine	93	91.2
Gynecology and Obstetrics	29	28.4
Pediatric and adolescent medicine	29	28.4
Psychiatry and Psychotherapy	24	23.5
Cardiology	17	16.7
Tropical and travel medicine	16	15.7
Dermatology	12	11.8
Endocrinology/Diabetology	12	11.8
Orthopedic surgery and Traumatology	12	11.8
Rheumatology	12	11.8
Surgery	11	10.8
Urology	11	10.8
Pediatric and adolescent psychiatry and psychology	9	8.8
Ophtalmology	9	8.8
Work Medicine	8	7.8
Medical Oncology	8	7.8
Radiology	8	7.8
Pneumonology	7	6.9
Prevention and healthcare	7	6.9
Allergiology	5	4.9
Oto-Rhino-Laryngology	5	4.9
Hand Surgery	4	3.9
Medical Genetics	4	3.9
Angiology	3	2.9
Hematology	3	2.9
Cardiac and thoracic vascular surgery	3	2.9
Infectiology	3	2.9
Neurosurgery	3	2.9
Neurology	3	2.9
Pathology	3	2.9
Plastic reconstructive and aesthetic surgery	3	2.9
Anesthesiology	2	2
Gastroenterology	2	2
Vascular Surgery	2	2
Pediatric Surgery	2	2
Clinical Pharmacology and Toxicology	2	2
Nephrology	2	2
Pharmaceutic Medicine	2	2
Physical medicine and rehabilitation	2	2
Forensic Medicine	2	2
Oral and maxillofacial surgery	1	1
Thoracic Surgery	1	1
